# Comparison of the seventh and eighth editions of the UICC/AJCC staging system for nasopharyngeal carcinoma: analysis of 1317 patients treated with intensity-modulated radiotherapy at two centers

**DOI:** 10.1186/s12885-018-4419-1

**Published:** 2018-05-29

**Authors:** Xing-Li Yang, Yan Wang, Shao-Bo Liang, Sha-Sha He, Dan-Ming Chen, Hai-Yang Chen, Li-Xia Lu, Yong Chen

**Affiliations:** 1 Department of Radiation Oncology, The First Affiliated Hospital of Sun Yat-sen University, Guangzhou, 510060 Guangdong People’s Republic of China; 2 Department of Radiation Oncology, Sun Yat-sen University Cancer Center, Guangzhou, China; 3 State Key Laboratory of Oncology in South China, Collaborative Innovation Center for Cancer Medicine, Sun Yat-sen University Cancer Center, Guangzhou, China; 4 Department of Radiation Oncology, Cancer Center, First People’s Hospital of Foshan Affiliated to Sun Yat-sen University, Foshan, China; 5 The Sixth Affiliated Hospital of Sun Yat-sen University, Guangzhou, China; 6 Department of Radiation Oncology, Collaborative Innovation Center for Cancer Medicine, State Key Laboratory of Oncology in South China, Sun Yat-sen University Cancer Center, Guangzhou, 510060 Guangdong People’s Republic of China

**Keywords:** Nasopharyngeal cancer, Staging system, Intensity-modulated radiation therapy, Prognostication

## Abstract

**Background:**

In the intensity-modulated radiotherapy (IMRT) era, great improvement has been made in survival of nasopharyngeal carcinoma (NPC). The 7th edition of the International Union against Cancer/American Joint Committee on Cancer (UICC/AJCC) staging system seems “outdated ” as it mainly based on the study in 2D/3D era, and thus the 8th edition has made some amendments according to recent studies. We aimed to compare and evaluate these two editions of staging system for NPC in patients treated with intensity-modulated radiotherapy.

**Methods:**

A total of 1317 patients with biopsy-proven, non-metastatic NPC treated with IMRT between 2009 and 2014 at two institutions were retrospectively assessed. All patients were assessed by magnetic resonance imaging and restaged according to the 7th and 8th editions. Prognostic factors for local relapse-free survival (LRFS), distant metastasis-free survival (DMFS), disease-free survival (DFS) and overall survival (OS) were assessed and compared using the Kaplan-Meier method and log-rank test. The Cox proportional hazards model was also used to calculate the hazard ratio (HR).

**Results:**

In both 7th and 8th edition, insignificant difference could be observed between T2 and T3 disease, T2 and T4 disease (all *P* > 0.05) for LRFS, while the difference of LRFS between T3 and T4 disease was significant in the previous edition (*P* = 0.001) but insignificant (*P* = 0.279) after revision. For OS, highly similar survival curve could be seen between T2 and T3 disease in both edition (all *P* > 0.1). DMFS and OS were not significantly different between N3a and N1-3b categories of the 7th edition (all *P* > 0.05). In contrast, obvious segregation was observed between N3 and the other N categories after the revision and combination in the 8th edition (all *P* < 0.05). DFS and OS were not significantly different between stage IVA and IVB of the 7th edition (*P* = 0.057 and *P* = 0.365, respectively); therefore, combining these stages in the 8th edition was reasonable.

**Conclusion:**

The overall stages and N categories of the 8th edition of the UICC/AJCC staging system provide better segregation of survival outcomes than the 7th edition. The 8th edition is also more clinically applicable as it has reduced ambiguity and revised out-of-date definitions. However, the T categories need further optimizing as the 8th edition failed to solve the problem of similar survival between adjacent T-classification, which has been exited since 7th edition.

## Background

Nasopharyngeal carcinoma (NPC) is mysterious malignancy with marked racial and geographical differences which was prevalent in Southern China, Southeast Asia and North Africa [1]. Crude incident in China had reached up to 3.09/100,000 in 2012 and the age-standardized incidence rates by world standard population (ASIRW) in south China is 9.69/100,000 [2]. The extensive use of magnetic resonance imaging (MRI) and intensity-modulated radiation therapy (IMRT) have markedly improved 5-year survival rates in NPC, especially local relapse-free survival (LRFS), which now exceeds 90% [3, 4].

The TNM staging system developed by the International Union against Cancer (UICC) and American Joint Committee on Cancer (AJCC) is considered the authoritative system for assessing disease progression, predicting prognosis and assisting treatment selection [5–7]. Therefore, the importance of accurate staging in terms of selecting and determining treatment strategies cannot be overemphasized. Since the 7th edition of the UICC/AJCC staging system had been internationally recommended, numerous studies confirmed its ability to predict prognosis [8–10]. However, the use of ambiguous or out-of-date definitions limited the clinical relevance of the 7th edition [11–13]. Fortunately, there is improvement of this aspect in the 8th edition. Firstly, the ambiguous definition-infratemporal fossa (ITF)/masticatory space (MS), which was regarded as T4 in the 7th edition, has been replaced by a more specific description in the 8th edition—the MP, LP and prevertebral muscles are included as T2, and the parotid gland and lateral surface of the LP muscle as T4 [14]. Secondly, widespread use of MRI in diagnosis and IMRT in treatment calls for a cross-sectional imaging method to replace supraclavicular fossa (SCF), which was primarily based on clinical examination and treated as the boundary for N3b to other N stage disease in the previous edition. In the 8th staging edition, such demoded term was replaced by lower level (LL), which is defined as the area below the caudal border of the cricoid cartilage. Moreover, there are other revisions incorporated into the 8th edition of the UICC/AJCC staging system for NPC. In the T category classification, EBV-positive cervical nodes were added as T0 disease, the prevertebral muscle invasion was added as T2, and the cervical vertebra invasion was added as T3. In the N category classification, N3a and N3b in the 7th edition were merged to N3. For clinical stage, stages IVA and IVB of the 7th edition were merged into stage IVA; Correspondingly, previous stage IVC was upgraded to stage IVB in the new edition [15].

Although the revisions incorporated into the 8th edition were based on a large-sample study from two centers and supported by evidence from multiple centers [15], the prognostic value of the 8^th^ edition needs to be validated using data from other centers. In this analysis, 1317 patients with NPC without distant metastasis at diagnosis who received IMRT with or without chemotherapy at two institutions were assessed to compare the prognostic performance of the 7th and 8th editions of the UICC/AJCC staging system.

## Methods

The study protocol was designed in accordance with the guidelines outlined in the Declaration of Helsinki and was approved by the Ethics Committee of the First Hospital of Foshan and Sun Yat-Sen University Cancer Center (South China). The requirement for informed consent was waived due to the retrospective nature of the study.

### Patient characteristics

A total of 1317 eligible patients (1014 males and 303 females; median age, 47.3 years; range, 13–83) with NPC treated at the First Hospital of Foshan (776 patients, 58.8%) or Sun Yat-Sen University Cancer Center (541 patients, 41.2%) between October, 2009 and March, 2014 were retrospectively enrolled using the same inclusion criteria: (i) patients with pathological evidence of NPC; (ii) with complete baseline clinical information and laboratory data; (iii) who received IMRT; and (iv) with complete follow-up data. Patients with distant metastasis at presentation were excluded.

According to the 2003 World Health Organization (WHO) classification, 99.8% of all patients had non-keratinizing carcinoma and the remainder (0.2%) had basaloid squamous cell carcinoma. The tumor, node and stage distributions of the 1317 patients according to the 7^th^ and 8th editions are presented in Tables 2, 3 and 4.

### Treatment

All patients were treated with IMRT at a median total dose of 70 Gy (range, 63–76 Gy) in 31 fractions (range, 28–36 fractions) at 2.26 per fraction to the planning target volume (PTV) of the gross primary tumor volume and 68 Gy (range, 50–75 Gy) in 31 fractions (range, 20–35 fractions) to the PTV of the gross nodal tumor volume (GTV-N), 60 Gy in 31 fractions to the PTV of the high-risk clinical target volume (CTV1), and 54 Gy in 31 fractions to the PTV of the low-risk clinical target volume (CTV2). All patients received one fraction daily, 5 days per week. Overall, 88 (6.7%) patients received additional intracavitary irradiation for tumor persistence.

According to institutional guidelines, chemotherapy was recommended for patients with stage II-IVB NPC (7th edition). Overall, 87.4% (1151/1317) of patients received chemotherapy. Concomitant chemotherapy was delivered to 918 patients: 211 with stage II and 703 with stage III to IVB NPC (7th edition); 505 patients with stage II to IVB disease received both induction and concomitant chemotherapy; no patients received adjuvant chemotherapy. In total, 92.0% (451/490) of patients with stage III NPC and 93.2% (419/440) of patients with stage IVA and IVB NPC received chemotherapy. Neoadjuvant chemotherapy consisted of cisplatin (80 mg/m^2^) and fluorouracil (1000 mg/m^2^ daily for 4 days); docetaxel (75 mg/m^2^) and cisplatin (75 mg/m^2^); or a triplet of docetaxel (60 mg/m^2^), cisplatin (60 mg/m^2^) and fluorouracil (800 mg/m^2^ daily for 4 days) every 3 weeks for 2–3 cycles. Concurrent chemotherapy was cisplatin given every 3 weeks (100 mg/m^2^or 80 mg/m^2^) or weekly (40 mg/m^2^) during RT.

### Follow-up and statistical analysis

After treatment, all patients were assessed every 3 to 6 months in the first 3 years, then every 6 to 12 months. Follow-up was calculated from the first day of treatment until death or last examination visit. June 27th, 2017 was the last follow-up date. Median follow-up was 55.62 months (range; 1.47–90.17 months).

Statistical Package for the Social Sciences (SPSS) software, version 20.0 (SPSS, Chicago, IL USA) was used to perform analysis. Actuarial rates were estimated using the Kaplan-Meier method [16], and survival curves were compared using the log-rank test [17]. All endpoints: local relapse-free survival (LRFS), distant metastasis-free survival (DMFS), disease-free survival (DFS) and overall survival (OS), were defined as the interval to the first defining event. Multivariate analyses with the Cox proportional hazards model were used to test the independent significance of different parameters by forward elimination of insignificant explanatory variables. The Cox proportional hazards model was also used to calculate the hazard ratio (HR). A two-tailed *P*-value < 0.05 was considered statistically significant.

## Results

As a result of the revisions in the 8th edition (Table. 1), 8/1317 (0.6%) patients in this cohort were up-staged from T1 to T2, 19/1317 (1.4%) were down-staged from T4 to T2, 116/1317 (8.8%)were down-staged from T4 to T3, and 12/1317 (0.9%) and 26/1317 (2.0%) patients were upstaged to N3 from N1 and N2, respectively. In terms of overall stage, 2/1317 (2%) patients were up-staged from stage I to II, 9/1317 (0.7%) from II to IVA and 17/1317 (1.3%) from III to IVA, and 10/1317 (0.8%) and 113/1317 (8.6%) patients were down-staged from IVA to II and III, respectively (Tables 2, 3 and 4).

**Table 1:** Classification criteria of the 7th and 8th editions of the UICC/AJCC staging system for nasopharyngeal carcinoma

	7^th^ edition	8^th^ edition
T category		
T0		No tumor identified, but EBV-positive cervical node involvement
T1	Nasopharynx, oropharynx or nasal cavity	Nasopharynx, oropharynx, nasal cavity without parapharyngeal involvement
T2	Parapharyngeal extension	Parapharyngeal space and/or adjacent soft tissue involvement (medial pterygoid, lateral pterygoid, prevertebral muscles)
T3	Bony structures and/or paranasal sinuses	Bony structures at skull base, cervical vertebra, pterygoid structures, and/or paranasal sinuses
T4	Intracranial extension and/or cranial nerves, hypopharynx, orbit or infratemporal fossa/masticatory space*	Intracranial extension, involvement of cranial nerves, hypopharynx, orbit, parotid gland and/or extensive soft tissue infiltration beyond lateral surface of lateral pterygoid
N category		
N0	None	None
N1	Unilateral cervical and/or unilateral or bilateral retropharyngeal node(s), ≤ 6 cm in greatest dimension, above supraclavicular fossa	Unilateral cervical and/or unilateral or bilateral retropharyngeal node(s), ≤ 6 cm in greatest dimension, above caudal border of cricoid cartilage
N2	Bilateral cervical node(s), ≤ 6 cm in greatest dimension, above supraclavicular fossa	Bilateral cervical node(s), ≤ 6 cm in greatest dimension, above caudal border of cricoid cartilage
N3	N3a > 6 cm in greatest dimension, above supraclavicular fossa	Unilateral or bilateral cervical node(s), > 6 cm in greatest dimension, below caudal border of cricoid cartilage
	N3b in supraclavicular fossa	
Clinical stage		
I	T1N0M0	T1N0M0
II	T1N1M0, T2N0-1M0	T1N1M0, T2N0-1M0
III	T1-2N2M0, T3N0-2M0	T1-2N2M0, T3N0-2M0
IV	IVA: T4N0-2M0	T4N0-2M0, T0-4N3M0
	IVB: any T1-4N3M0	

^a^infratemporal fossa/masticatory space: the superficial layer of the deep cervical fascia splits to enclose the four masticatory muscles, including the medial pterygoid (MP), lateral pterygoid (LP), temporalis and masseter muscles, to enclose this space

**Table 2:** Distribution of T categories as defined by the 7th and 8th editions

		8th edition				
		T1	T2	T3	T4	Total
7th edition	T1	324 (24.6%)	8 (0.6%)			332 (25.2%)
	T2		166 (12.6%)			166 (12.6%)
	T3			420 (31.8%)		420 (31.8%)
	T4		19 (1.4%)	116 (8.8%)	264 (20.0%)	399 (30.3%)
	Total	324 (24.6%)	193 (14.6%)	536 (40.7%)	264 (20.0%)	1317

**Table 3:** Distribution of N categories as defined by the 7th and 8th editions

		8th edition				
		N0	N1	N2	N3	Total
7th edition	N0	250 (19.0%)				250 (19.0%)
	N1		719 (54.6%)		12 (0.9%)	731 (55.4%)
	N2			252 (19.1%)	26 (2.0%)	278 (21.1%)
	N3a				16 (1.2%)	16 (1.2%)
	N3B				42 (3.2%)	42 (3.2%)
	Total	250 (19.0%)	719 (54.6%)	252 (19.1%)	96 (7.3%)	1317

**Table 4:** Distribution of overall stage as defined by the 7th and 8th editions

		8th edition				
		I	II	III	IVA	Total
7th edition	I	93 (7.1%)	2 (0.2%)			95 (7.3%)
	II		283 (21.5%)		9 (0.7%)	292 (22.2%)
	III			473 (35.9%)	17 (1.3%)	490 (37.2%)
	IVA		10 (0.8%)	113 (8.6%)	259 (19.7%)	382 (29.0%)
	IVB				58 (4.4%)	58 (4.4%)
	Total	93 (7.1%)	295 (22.4%)	586 (44.5%)	343 (26.0%)	1317

### Patterns of failure and survival outcomes

In total, 75/1317 (5.7%) and 171/1317 (13.0%) patients developed local recurrence and metastasis, and 198 (15.0%) died. The median time to local and distant failure was 26.17 (range: 7.00–69.37) and 21.96 (range: 1.9–69.13) months, respectively. The 4-year LRFS, DMFS, DFS, and OS rates were 94.4%, 87.3%, 82.1%, and 87.6%, respectively.

### T classification

**Fig. 1:**
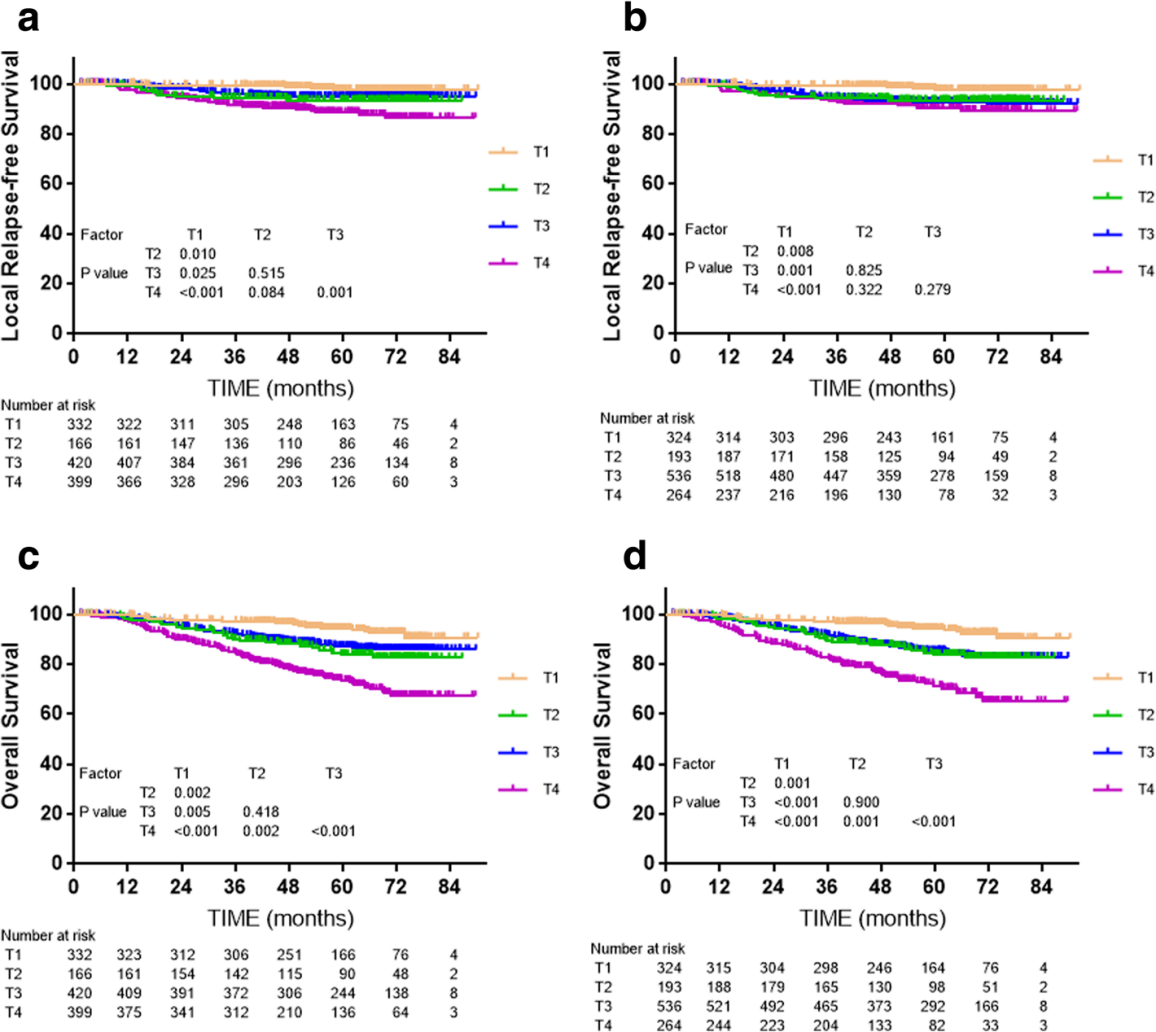
Survival analyses for the T category classifications of the 7th and 8th edition staging systems. **a** and **c**: Local relapse-free survival and overall survival for T categories defined by the 7th edition; **b** and **d**: local relapse-free survival and overall survival for T categories defined by the 8th edition

Cox multivariate regression analysis showed the T category classifications of both editions were independent prognostic factors for LRFS and OS (*P* < 0.001). For the 7th edition, LRFS and OS were not significantly different between T2 and T3 (*P* = 0.515 and *P* = 0.418, respectively, Fig. 1), while LRFS was borderline significantly different between T2 and T4 (*P* = 0.084, Fig. 1), with a clear distinction in OS between T2 and T4 (*P* = 0.002, Fig. 1). However, no significant differences in LRFS were observed between T2 and T3, T2 and T4, and T3 and T4 of the 8th edition (*P* = 0.825, *P* = 0.332 and *P* = 0.279, respectively, Fig. 1), and the OS curves for T2 and T3 of the 8th edition even overlapped (*P* = 0.900, Fig. 1). In summary, the T categories of the 8th edition seems failed to raise obviously superior prognostic value compared to the 7th edition.

**Fig. 2:**
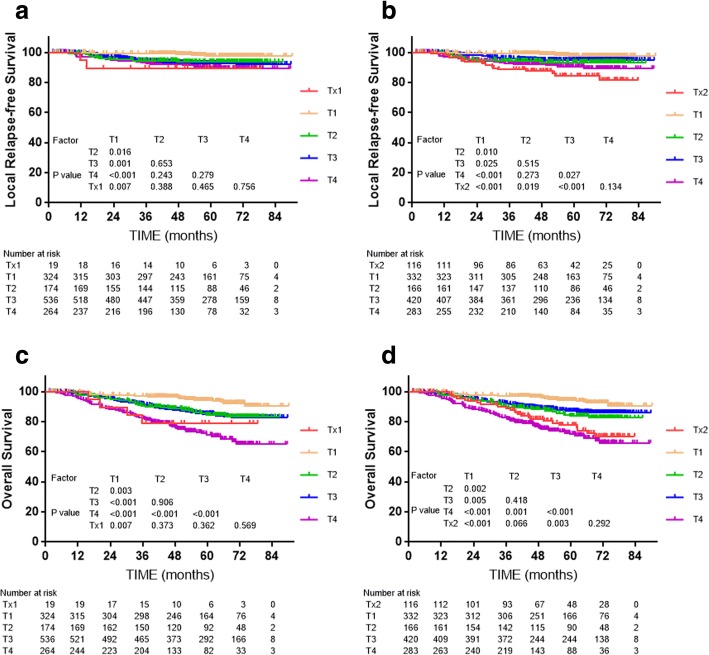
Survival analyses for the masticatory space subset compared with other subsets using the 7th edition of the staging system. **a** and **b**: Local relapse-free survival and overall survival for MSI without other T3/T4 criteria; **c** and **d**: Local relapse-free survival and overall survival for MSI without other T4 criteria

Subgroup analysis was conducted to explore the prognostic significance of MP and LP involvement. 19 patients with MP/LP invasion who did not fulfill the criteria for T3 or T4 in the 7th edition, and who were restaged as Tx1 in the 8th edition, did not have significantly different LRFS compared to T2, T3 or T4 of the 7th edition (*P* = 0.388, *P* = 0.465 and *P* = 0.756, respectively, Fig. 2). The 116 patients with T3 tumors with anatomical MP/LP involvement, who were staged as T4 in the 7th edition but as T3 in the 8th edition, had similar survival to T4 and significantly different survival to T3 without anatomical MP/LP involvement for the 7th edition (*P* < 0.001, Fig. 2).

Due to the lack of differences in LRFS between the T categories of the 8th edition, multivariate Cox regression analysis was performed to evaluate the various prognostic factors used to define the T categories (Table 5). The following covariables were included in the Cox proportional hazards model with backward conditional: age (≤ 50 vs. > 50 years), gender (female vs. male), paranasal sinus, skull base infiltration, medial pterygoid muscle, lateral pterygoid muscle, prevertebral muscle, 8th edition N category, and chemotherapy (no vs. yes). Multivariate Cox regression analyses showed that prevertebral muscle extension and medial pterygoid muscle extension were the independent factors for local failure. Gender, prevertebral muscle extension, cranial nerve invasion and N category of 8th edition were significantly associated with disease failure. Notably, male and advanced N category may more likely to develop disease failure. Male, aged (> 50 years old), prevertebral muscle extension, skull base infiltration, cranial nerve invasion and more advanced N category of 8th edition were found to be the independent factors for OS (Table. 5).

**Table 5:** Independent prognostic survival factors for local relapse, disease failure and death in multivariate Cox regression analyses

End-point	Factor	Value	HR	95% HR
Local failure	Prevertebral muscle	0.048	1.319	1.003–1.734
	Medial pterygoid muscle	0.011	1.607	1.116–2.315
Disease failure	Gender (female vs. male)	0.012	1.539	1.100–2.154
	Prevertebral muscle	< 0.001	1.339	1.171–1.532
	Cranial nerve	< 0.001	1.675	1.291–2.171
	8th edition N category	< 0.001	1.692	1.461–1.960
Death	Gender	0.007	1.715	1.162–2.530
	Age (≤ 50 vs. > 50)	0.031	1.365	1.028–1.817
	Prevertebral muscle	0.001	1.308	1.111–1.541
	Skull base infiltration	0.011	1.582	1.113–2.248
	Cranial nerve	< 0.001	1.860	1.454–2.379
	8th edition N category	< 0.001	1.615	1.362–1.191

*HR* hazard ratio, *CI* confidence interval

### N classification

**Fig. 3:**
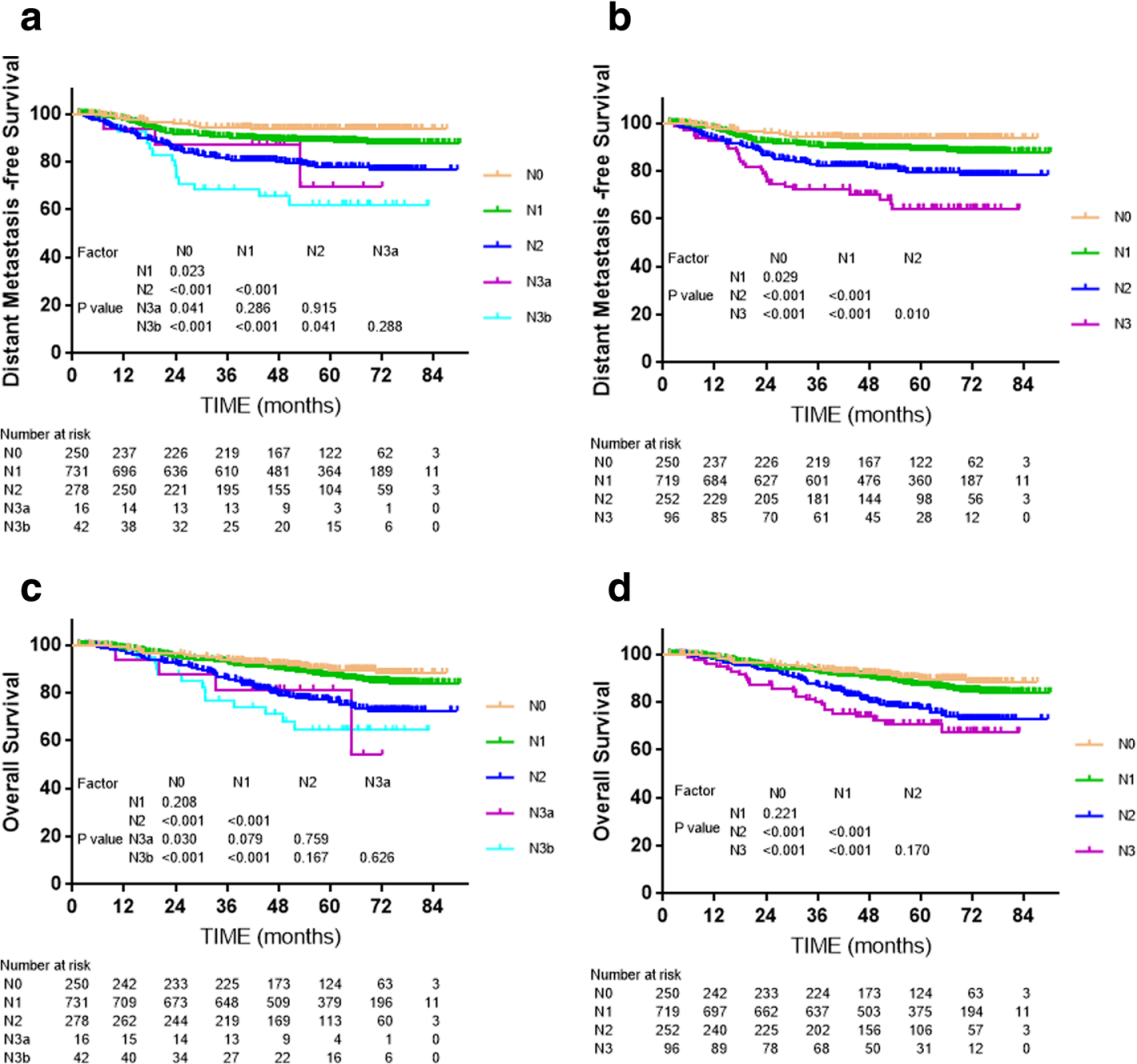
Survival analyses for the N category classifications of the 7th and 8th editions of the UICC/AJCC staging system. **a** and **c**: Distant metastasis-free survival and overall survival for the N categories of the 7th edition; Panels **b** and **d**: Distant metastasis-free survival and overall survival for the N categories of the 8th edition

Cox multivariate regression analysis showed that the N category classifications of both editions were independent prognostic factors for local recurrence free survival and overall survival (*P* < 0.001, Fig. 3). For example, significant separations in distant metastasis-free survival (DMFS) were observed between adjacent N categories of the 8th edition (Fig. 3), but the differences between N3a and N1, N2, N3b of the 7th edition were insignificant (*P* = 0.286, *P* = 0.915, *P* = 0.288, respectively, Fig. 3). The small number of patients with N3a (*n* = 16) disease may have reduced statistical power. Moreover, the differences in OS between N0 and N1, and N2 and N3 (including N3a and N3b in the 7th edition) were not significant (all *P* > 0.05, Fig. 3), though the difference between N0 and N1 was slightly larger for the 8th edition than 7th edition.

**Fig. 4:**
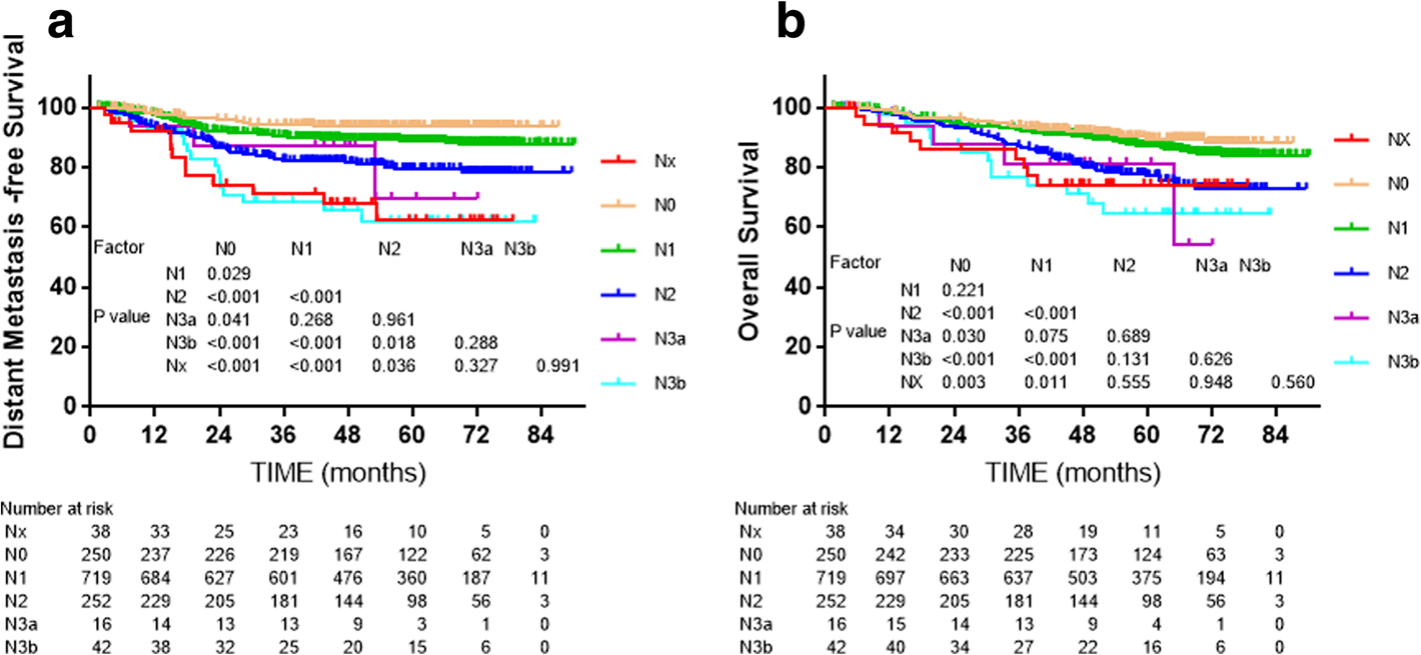
Survival analyses for the lower neck subset compared with other subsets using the 7th edition of the UICC/AJCC staging system. **a** and **b**: Distant metastasis-free survival and overall survival for the change from the supraclavicular fossa in the 7th edition to the lower neck in the 8th edition

Subgroup analysis was conducted to assess the value of altering SCF to the lower neck in the 8th edition. Patients upstaged from N1 and N2 in the 7th edition to N3 in the 8th edition were staged as NX (lymph nodes above SCF but below the caudal border of the cricoid cartilage) in the subgroup analysis; the other criteria were the same as the 7th N categories, apart from the lower neck alteration. No statistically significant differences in 4-year DMFS and OS were detected between Nx and N3a or N3b (*P* = 0.288, *P* = 0.991, respectively, Fig. 4).

### Overall stage

**Fig. 5:**
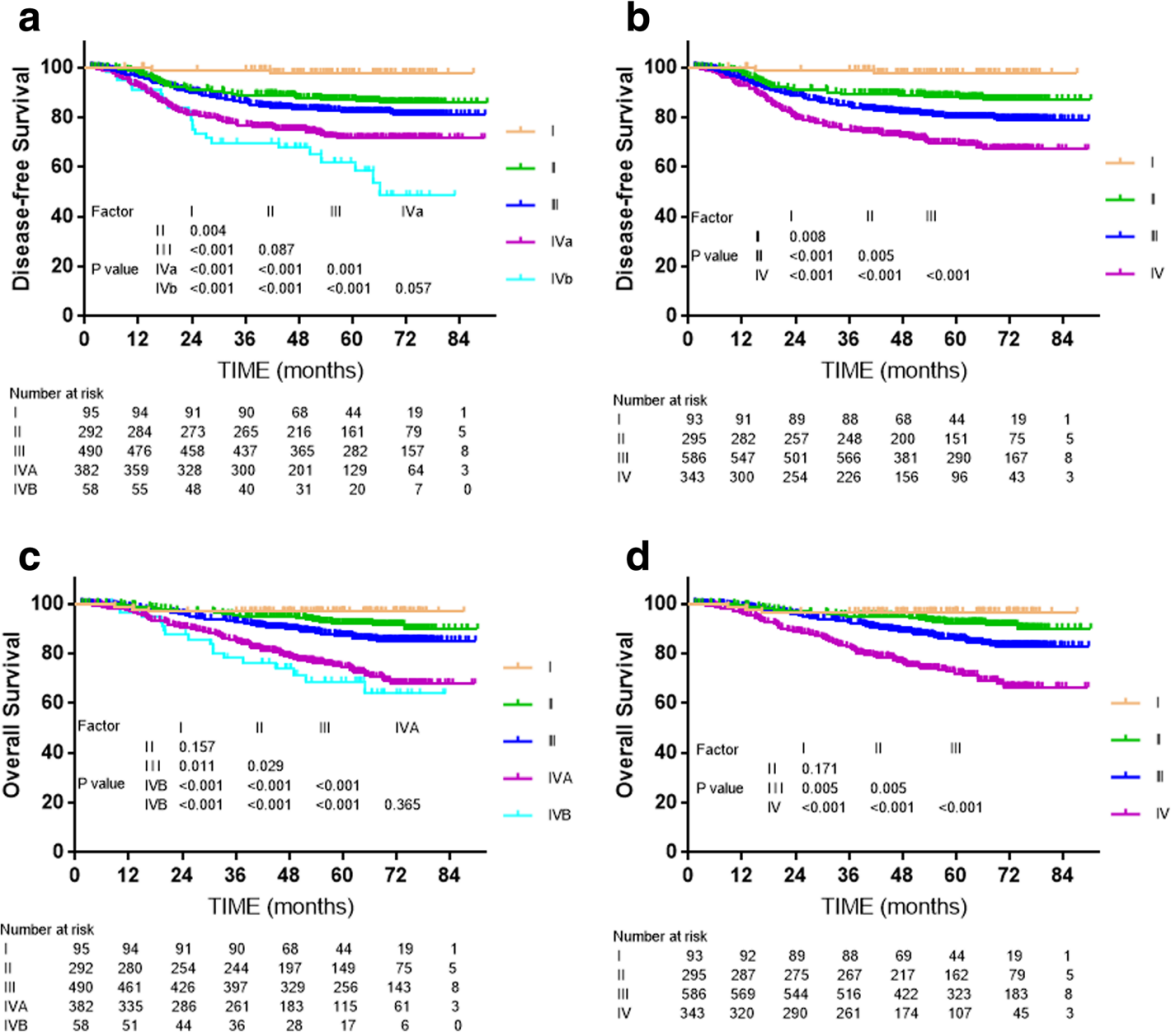
Survival analyses for the overall stages of the 7th and 8th editions of the UICC/AJCC staging system. **a** and **c**: Disease-free survival and overall survival for the T categories of the 7th edition; **b** and **d**: Disease-free survival and overall survival for the T categories of the 8th edition

The overall stage classifications of both editions were independent prognostic factors for death, disease failure, local failure and disease failure in Cox multivariate regression analysis (*P* < 0.001, Fig. 5). For DFS and OS, the differences between stage IVA (T4 N0–2) and IVB (T1-3 N3) of the 7th edition were not significant (*P* = 0.057, *P* = 0.365, respectively, Fig. 5); therefore, it was reasonable to merge T4 and N3 disease. Compared to the 7th edition, the 8th edition provided better segregation of long-term DFS and OS between adjacent clinical stages (Fig. 5).

## Discussion

The UICC/AJCC TMN staging system is the authoritative method of assessing the extent of local invasion, regional lymphatic spread and distant metastasis, and is considered the most valuable prognostic factor in NPC. Although the 8^th^edition was only published one year ago, several studies have attempted to validate its clinical applicability. Pan et al. reported clear separation was not observed between stage I and II (*P* = 0.07 and *P* = 0.10, respectively) of the 7th and 8th editions. Tang et al. [18] and Xu et al. [19] found no significant differences between stage II and III (all *P* > 0.05) of either edition. However, overlapping between these cohorts was inevitable, as the patients were from the same center and treated during the same period [21, 22]. OuYang et al. [20] compared the proposed Guangzhou, Hong Kong, Guangxi staging system with the 7th and 8th editions of the AJCC/UICC staging system using a cohort of 899 patients. They found the N classification of the 8th edition had better prognostic performance than the 7th, while the T category classification still required further optimization. In this study, a total of 1317 patients treated with IMRT at two different hospitals were assessed to compare the prognostic value of the 7th and 8th editions of the UICC/AJCC staging system.

### Study limitations

Firstly, this study was a retrospective study of 1317 patients from two centers in Guangdong Province of China. In some subgroups, especially the subgroups with PSI and MSI, a small number of patients limited the reliability of our conclusions. Larger-scale analyses are needed to confirm this study. Secondly, only 372 (28.2%) patients received PET-CT before treatment. Thirdly, other factors such as EBV DNA [21] and primary tumor volume [22], which have been found to have a profound influence on prognosis, were not considered in this study.

### T classification

In a study which compared different staging systems including the 7th, 8th edition of AJCC/UICC staging system and Guangzhou, Hongkong, Guangxi staging system, Guangzhou staging system led to the highest c-index in T classification and the 8th edition ranked the second [20]. Minor difference was found between these two systems, which was extension of Oropharynx or nasal cavity was staged as T1 disease in the 8th edition but T2 in Guangzhou system. Nevertheless, validation between 7th and 8th edition of AJCC/UICC staging system from the same center showed c-index in the previous edition was slightly higher than the latest one [18].

In this study, the 8th edition failed to solve the problem of similar survival between adjacent T-classification, which has been exited since 7th edition; indeed, the lack of significance between T categories was more obvious for the 8th edition, which mainly own to the alteration of ITF/MS. In fact, IFT/MS involvement has long been included in the UICC/AJCC staging system as a T4 criterion, though the exact anatomical boundaries for these structures have varied between editions [23, 24]. In the 5th and 6th editions, the ITF/MS did not involve the medial pterygoid (MP) or lateral pterygoid (LP) [25], while the 7th edition definition of the MS included all four masticatory muscles: MP, LP, temporalis and masseter [14]. It was laudable that descriptions in the latest edition were more specific. However, the best classification of IFT /MS had not reach a consensus. Pan et al. found patients without T3 or T4 criteria but MP/LP involvement achieved much better 5-year OS than patients with T4 disease with other criteria except for MP/LP involvement (93% vs. 71%, respectively, *P* = 0.003) [15]. A similar result was reported by Tang et al. [26], though different degrees of MS invasion did not significantly affect LRFS or OS (*P* = 0.34 and *P* = 0.54, respectively). In another study of 816 patients, including 283 (36.4%) patients with MS invasion, MS involvement was an independent prognostic factor for local control (*P* = 0.007) and OS (*P* = 0.024) in multivariate analyses, and patients with MP involvement had similar survival rates as T2 or T3 disease (all *P* > 0.1), though the outcomes for patients with LP involvement were similar to T4 disease (*P* > 0.1) [27]. In this study, limited number of patients in the subgroup showed that MS involvement with T3 criteria had similar survival outcomes to T4 disease in this study (*P* = 0.134 for LRFS, *P* = 0.292 for OS). Such discrepancies may be due to the varied demographics, inclusion criteria, treatment strategies and follow-up times in each study, and the a larger-scale, multicenter study is wanted to figure the staging of MS.

Involvement of the prevertebral muscles, mentioned for the first time in the 8th edition as a T2 criterion, has been shown to increase the risk of local and distance failure. In a study of 506 patients, prevertebral space invasion (PSI) was associated with similar survival to T4 disease, but not T3 [28]. However, due to the lack of a significant difference in OS between PSI and MS invasion reported by Pan et al. [15], single PSI was classified as T2 in the 8th edition. Unfortunately, only eight patients had PSI without T2, T3 or T4 criteria in this study; this sample size was too small conduct subgroup analysis. However, multivariate analysis showed PSI was independent prognostic factor for LRFS, DFS and OS. More detailed studies of a lager cohort are required.

The marginal differences in prognosis between adjacent T categories of the 7th and 8th editions (Fig. 1) reflect developments in diagnosis and treatment. On the one hand, the widespread use of MRI makes skull base erosion easier to detect [3, 29]. Although MRI can more precisely detect deep tumor infiltration and has improved LRFS by around 20% [30], some early micro-migration—which can only be detected by MRI—may have a better prognosis than the obvious invasion easily observed on CT scans in other patients with the same T category. Compared to the erosion easily detected on CT, skull-base erosion detectable on MRI but undetectable on CT may have a more favorable prognosis [30, 31]. On the other hand, the popularity of IMRT and addition of chemotherapy have also reduced local failure [32]. Distant metastasis remains the main failure pattern in NPC, further emphasizing the importance of accurate N category classification.

### N classification

In Ouyang’s study, which compared five staging systems, N-classification in the 8th edition of AJCC/UICC staging system owned higher C-index for OS, DMFS and RRFS than the previous edition [20]. Another validation of the 8th edition also supported that the new prognostic model of N-classification predicted outcomes fairly well [18].

Compared to the N category classification of the 7th edition, the 8th edition became consistent with the consensus guidelines used for other head-and-neck cancers [33], making the staging system more convenient in clinical practice, and also resulting in better segregation of both DMFS and OS (Fig. 2).

The 8th edition uses the caudal border of the cricoid cartilage to differentiate N1–2 and N3 [15], in other words, the LL is a demarcating criterion for N3. The data supporting the proposal of the 8th edition did not show this replacement improved prognostic value, though there was little controversy about the alternation. SCF, defined by the superior margin of the sternal end of the clavicle, the superior margin of the lateral end of the clavicle and the point where the neck meets the shoulder [34], is not a reliable radiological landmark in this IMRT era when MRI is widely used for diagnosis while the new boundary - lower level (LL), defined as the area below the caudal border of the cricoid cartilage -is an anatomical landmark that can be reliably defined on physical examination and also accurately located in cross-sectional images. Replacing the SCF with the LL is sensible and practicable as the LL corresponds to the entire area of levels IVa, IVb, Vb and Vc as defined by the 2013 International Consensus Guidelines [33]. Yue et al. found that, compared to Ho’s SCF, the LL provided more distinct separation of DMFS, DFS and OS between adjacent N categories [11]. A similar result was obtained in this study. Moreover, 38 patients (about 3%) in our cohort were upstaged from 7th edition N1 or N2 to 8th edition N3 because of this change, and these patients achieved closer survival outcomes to N3 than N1 or N2 (Fig. 4). Therefore, it is reasonable to assign lymph node(s) metastasis in the LL as a new N3 criterion.

Although Lee et al. [35] found maximal axial diameter (MAD) was a significant independent predictor of survival, other relevant studies such as Teo et al. [36] and Heng et al. [37] deemed the prognostic value of MAD was mainly due to the fact large lymph nodes are more frequent at lower nodal levels. Only 25 (1.9%) patients had lymph node(s) with a MAD larger than 6 cm, of whom eight had lymph node involvement extending to the SCF (7th edition N3b). Similar overlaps have also been reported in other studies [10, 15]. Furthermore, the similar DMFS and DFS rates for N3a and N3b indicate that this sub-category separation is unnecessary.

### Clinical stage

Stages IVA and IVB of the 7th edition were merged into stage IVA in the 8th edition, and naturally, previous stage IVC was upgraded to IVB. The differences in DFS and OS between IVA and IVB of the 7th edition were insignificant, whereas the overall stages of the 8th edition resulted in better separation of the DFS and OS curves. Although no significant difference in OS was observed between stage I and II in either the 7th and 8th editions (*P* = 0.157 and *P* = 0.171, respectively), the distinction between stage I and II is necessary as chemotherapy may benefit patients with stage II.

## Conclusion

The 8th edition of the UICC/AJCC staging system for NPC has superior prognostic value compared to the 7th edition, especially as the 8th edition N categories and overall stages. The 8th edition is also more clinically applicable as it has reduced ambiguity and revised out-of-date definitions. However, several issues, including the T category classification, need to be further evaluated in additional studies.

## Abbreviations

AJCC: American Joint Committee on Cancer
DFS: disease-free survival
DMFS: distant metastasis-free survival
IMRT: intensity-modulated radiotherapy
ITF: Infratemporal fossa
LL: Lower level
LP: Lateral pterygoid
LRFS: Local relapse-free survival
MAD: Maximal axial diameter
MP: Medial pterygoid
MRI: Magnetic resonance imaging
MS: Masticatory space
NPC: Nasopharyngeal carcinoma
OS: Overall survival
PSI: Prevertebral space invasion
SCF: Supraclavicular fossa
UICC: International Union against Cancer
